# A novel M phase blocker, DCZ3301 enhances the sensitivity of bortezomib in resistant multiple myeloma through DNA damage and mitotic catastrophe

**DOI:** 10.1186/s13046-020-01597-9

**Published:** 2020-06-09

**Authors:** Liangning Hu, Bo Li, Gege Chen, Dongliang Song, Zhijian Xu, Lu Gao, Mengyu Xi, Jinfeng Zhou, Liping Li, Hui Zhang, Qilin Feng, Yingcong Wang, Kang Lu, Yumeng Lu, Wenxuan Bu, Houcai Wang, Xiaosong Wu, Weiliang Zhu, Jumei Shi

**Affiliations:** 1 Department of Hematology, Shanghai Tenth People’s Hospital, Tongji University School of Medicine, 301 Yanchang Road, Shanghai, 200072 China; 2 CAS Key Laboratory of Receptor Research, Drug Discovery and Design Center, Shanghai Institute of Materia Medica, Chinese Academy of Sciences, 555 Zuchongzhi Road, Shanghai, 201203 China; 3 Tongji University Cancer Center, Tongji University, Shanghai, 200092 China

**Keywords:** Multiple myeloma, Drug-resistance, DNA damage response, Cell cycle

## Abstract

**Background:**

DCZ3301, a novel aryl-guanidino compound previously reported by our group, exerts cytotoxic effects against multiple myeloma (MM), diffused large B cell lymphoma (DLBCL), and T-cell leukemia/lymphoma. However, the underlying mechanism of its action remains unknown.

**Methods:**

We generated bortezomib (BTZ)-resistant cell lines, treated them with various concentrations of DCZ3301 over varying periods, and studied its effect on colony formation, cell proliferation, apoptosis, cell cycle, DNA synthesis, and DNA damage response. We validated our results using in vitro and in vivo experimental models.

**Results:**

DCZ3301 overcame bortezomib (BTZ) resistance through regulation of the G_2_/M checkpoint in multiple myeloma (MM) in vitro and in vivo*.* Furthermore, treatment of BTZ-resistant cells with DCZ3301 restored their drug sensitivity. DCZ3301 induced M phase cell cycle arrest in MM mainly via inhibiting DNA repair and enhancing DNA damage. Moreover, DCZ3301 promoted the phosphorylation of ATM, ATR, and their downstream proteins, and these responses were blocked by the ATM specific inhibitor KU55933.

**Conclusions:**

Our study provides a proof-of-concept that warrants the clinical evaluation of DCZ3301 as a novel anti-tumor compound against BTZ resistance in MM.

## Introduction

Multiple Myeloma (MM) is a malignant hematologic disease characterized by clonal proliferation of malignant plasma cells. In 2003, the Food and Drug Administration (FDA) approved bortezomib (BTZ) as a first-line drug for MM therapy [1, 2]. However, over the past decade, there has been an increase in the cases of drug-resistance and MM relapse following BTZ treatment [3]. Once a patient develops resistance, BTZ is rendered ineffective at the usual tolerable doses [4]. Since the safety margin of BTZ is very narrow, increasing its dose will significantly augment the risk of side effects. Thus, there is an urgent need to develop new therapeutic compounds to counter BTZ resistance in MM.

Chromosomal aberrations are one of the causative factors for cell chemoresistance. Chromosomal aberrations can lead to the inactivation of essential ‘housekeeping genes’ that are involved in the regulation of several important cellular functions, including gene transcription, DNA synthesis, DNA repair, cell cycle arrest, senescence, and apoptosis [5]. Therefore, targeting these mechanisms may improve the outcomes of drug resistance. Drug resistant cells always exhibit a robust activation of the DNA damage response (DDR) and a higher intrinsic DNA repair activity [6, 7]. Therefore, these cells can withstand the effects of DNA damaging drugs and can repopulate cancer tissues with therapy-resistant cells after treatment [8].

For decades, genotoxic drugs have been a mainstay in cancer therapy. These drugs may play a role in the inhibition of cell proliferation at all stages of the cell cycle [9]. There are two critical pathways that participate in the regulation of responses after DNA damage: one is the ataxia telangiectasia mutated (ATM)-checkpoint kinase 2 (CHK2)-P53 pathway. This pathway is mainly related to the G_1_/S phase transition [10]. The other is the ATM- and Rad3-related (ATR)-checkpoint kinase 1 (CHK1)-Cdc25 pathway. When DNA damage occurs, it plays a critical role during the intra-S phase or G_2_/M phase [11]. However, increasing evidence indicates that the ATM/CHK2 and ATR/CHK1 pathways may interact in response to DNA damage [12].

DCZ3301 is a novel aryl-guanidino compound synthesized by our group. We have previously reported that it exhibits strong anti-cancer activity in MM^13^, diffuse large B-cell lymphoma (DLBCL) [13] and T-cell leukemia/lymphoma [14]. Results from our studies have so far indicated that DCZ3301 possesses a strong ability to block cancer cells in the G_2_/M phase. Furthermore, it may affect several other cancer-associated pathways regulated by STAT3, NFκB, AKT, and ERK1/2. Moreover, DCZ3301 retains its anti-MM activity in the presence of exogenous cytokines (IL-6 or VEGF) or BMSCs [15]. Thus, we speculated that DCZ3301 may counter drug resistance in MM. However, the underlying mechanism of DCZ3301-mediated G_2_/M phase arrest in MM or DLBCL remains unknown. Besides, it is not known whether DCZ3301 blocks the tumor cells in the G_2_ or M phase. In this study, we tested the inhibitory effects of DCZ3301 on BTZ-resistant MM cells in vitro and in vivo*,* and tried to elucidate the underlying mechanism of DCZ3301-mediated G_2_/M phase arrest. Our results showed that DCZ3301 treatment activated the ATM-ATR-CHK1 signaling pathway and restored the sensitivity of BTZ-resistant cells.

## Materials and methods

### Reagents

**Fig. 1:**
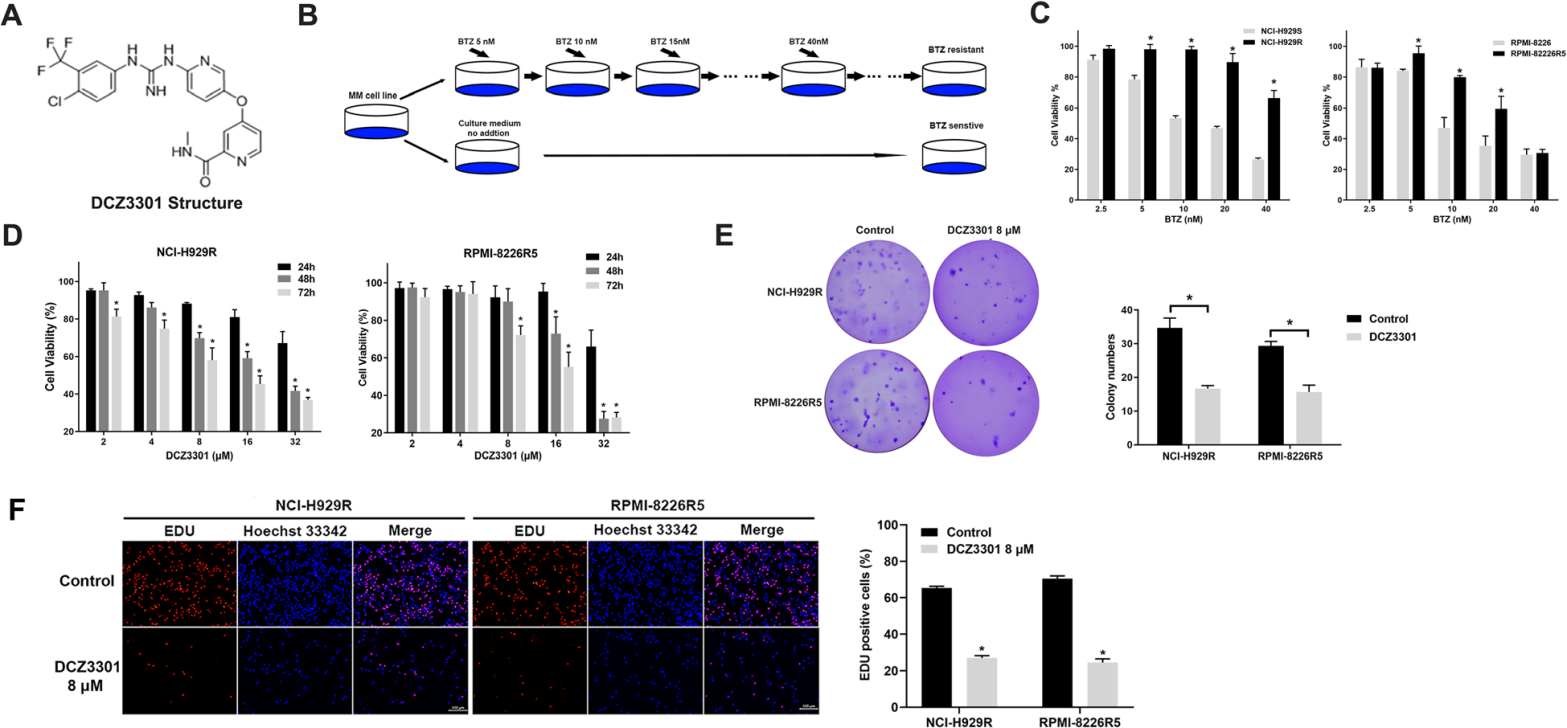
DCZ3301 treatment countered BTZ resistance and exhibited potent cytotoxicity against BTZ-resistant MM cells. (**a**) Molecular structure of DCZ3301. (**b**) The process of establishing BTZ-resistant cell lines. (**c**) Both BTZ-sensitive and BTZ-resistant MM cells treated with BTZ for 48 h and cell viability determined by CCK-8 assay. (**d**) CCK-8 assay demonstrated that DCZ3301 inhibited the viability of BTZ-resistant MM cells. (**e**) Soft agar colony formation by NCI-H929R and RPMI-8226R5 cells after DCZ3301 treatment. Representative images of colonies are shown in the left panel. Quantification of the colony numbers is presented in the right panel. (**f**) The effect of DCZ3301 on BTZ-resistant MM cell proliferation was evaluated by EdU incorporation assay. Scale bars = 100 μm.* *p* < 0.05 compared to control group. Data are presented as mean ± SD

DCZ3301 was kindly provided by Weiliang Zhu (Shanghai Institute of Materia Medica, Chinese Academy of Sciences, Shanghai, China) and the molecular structure is as shown in Fig. 1a with molecular weight of 464.0. DCZ3301 was stored at − 20 °C in DMSO (Sigma, St. Louis, MO) and the concentration of stock solution was 40 mM. Panobinostat was purchased from Selleck Chemicals (Houston, TX, USA). BTZ was obtained from Sigma (St. Louis, MO, USA). ATM kinase inhibitor KU55933 was obtained from Targetmol (Boston, MA, USA).

### Cell lines and culture condition

NCI-H929 and HEK-293 T were bought from American Type Culture Collection (ATCC) (Manassas, VA, USA). The BTZ-resistant MM cell line RPMI-8226R5 and the BTZ-sensitive cell line RPMI-8226 were kindly provided by Fenghuang Zhan (Department of Internal Medicine, University of Iowa, Iowa City, IA, USA). The BTZ-resistant cell line NCI-H929R was obtained by increasing extracellular concentrations of BTZ over a period of 10 months step by step (Fig. 1b). The BTZ-sensitive cell lines were routinely cultured in RPMI-1640 medium containing 10% fetal bovine serum (FBS), 100 IU/mL penicillin and 100 μg/mL streptomycin (GIBCO, Grand Island, NY), 5% Carbon dioxide. The BTZ-resistant cell lines were cultured in the presence of 20 nM BTZ in order to maintain the characteristics of BTZ resistance. And the BTZ was stopped adding for at least two weeks before every experiment. HEK-293 T cell line was cultured in DMEM/HIGH GLUCOSE medium (Gibco, Carlsbad, CA, USA). Cell lines were certificated by Short Tandem Repeat profiling (Shanghai Biotechnology Co., Ltd., Shanghai, China).

We used Ficoll-Hypaque density gradient centrifugation in order to gain the primary human cell lines. Primary CD138^+^ MM cells were selected from the bone marrow mononuclear cells (BMMCs) derived from MM patients. We stained these cells using human APC conjugated anti-CD138^+^ antibody (BioLegend, Sandiego, CA, USA). Blood samples were collected from healthy volunteers and Ficoll-Hypaque density gradient centrifugation was used to obtain peripheral blood mono-nuclear cells (PBMCs). In compliance with Declaration of Helsinki, written informed consent was obtained from MM patients and healthy donors. This study was approved by the institutional review board of the Shanghai Tenth People’s Hospital, Tongji University.

### Cell viability and apoptosis assay

Cells were seeded in 96-well plates at a density of 2 × 10^5^ cells/ml. After 24, 48 or 72 h treatment of DCZ3301, cell viability was assessed by using CCK8 kit (Yeasen Biotech, Shanghai, China). The half maximal inhibition concentration (IC_50_) was determined by using the CalcuSyn software program (CalcuSyn; Biosoft, USA). Alternatively, cells were treated with DCZ3301, BTZ and/or Z-VAD-FMK (50 μM, Selleckchem, Houston, TX) and then cell apoptosis was assessed by flow cytometry with Annexin V-FITC and PI (BD Pharmingen, San Diego, CA). The percentage of apoptosis in CD138^+^ primary MM cells was evaluated by staining Annexin-V and 7-aminoactinomaycin D (7-AAD, KeyGen Biotech, China). All percentage of cell apoptosis was measured by flow cytometer FACS Canto II flow cytometer (BD Bioscience, California, USA).

### Soft agar assay of colony formation

2000 cells mixed with RPMI1640 media containing 20% FBS and 0.33% agar were layered on top of the base layer of 0.5% agar. And we seeded these cells in 12-well plates. Then we use digital camera to take pictures of the colony. Image J 1.51j8 software (Wayne Rasband National Institutes of Health, Washington, USA) was used to scan and count the overall numbers of colony in the pictures.

### Cell cycle analysis

After treatment of DCZ3301, we collected the MM cells and resuspended the cells in 200 μl PBS. Cold 70% ethanol was used to fix cells morphology and then cells were washed in PBS. At last cells were dyed using 500 μl of PI/RNase staining buffer (BD, Pharmingen, Franklin Lakes, NJ, USA). In order to detect the percentage of M phase arrest cells, the cells were stained in 100 μL cell staining buffer with 3 μL Alexa Fluor®488-conjugated anti-phospho (Ser10)-Histone H3 polyclonal antibody (BioLend Inc., San Diego, CA, USA) for 30 min at room temperature according to the instruction. After staining, cells were detected using flow cytometry FACS Canto II flow cytometer (BD Bioscience, California, USA).

### Neutral comet assay

Comet assays were conducted following protocols provided by Comet Assay® Kit (Trevigen, Gaithersburg, USA). After treated with DCZ3301, cells were collected. 1 × 10^5^ cells/ml cells were combined with LMA agarose. Cell lysis was neutralized in neutral electrophoresis buffer for 30 min and then run for 45 min at 21 V. Cells then were sunk with DNA precipitation buffer and washed using 70% ethanol. Slides were dried at 37 °C before staining with SYBER green. Finally, the length of comet was observed using fluorescence microscopy (Leica, Wetzlar, Germany). CaspLab Comet Assay Software (CaspLab, Wroclaw, Poland) was used to evaluate the comet length of the relative cells.

### Immunofluorescence assay

2 × 10^5^ cells/ml cells were seeded in 6-well plates, and then administrated with DCZ3301 for 48 h. Then cells were harvested and fixed in 4% paraformaldehyde for 30 min. After washed in PBS, 3% BSA was used to block cells. And then permeated with 0.1% TritonX-100, and incubated γ-H2A.X (1:250 dilution; Abcam, Cambridge, MA, USA) or α-tublin (1:500 dilution; Sigma-Aldrich, St. Louis, USA) at 37 °C for 1 h. The secondary antibody Dylight™ 488-conjugated Affinipure Donkey antibody (1:400 dilution; Jackson Immuno Research Laboratory, USA) was incubated with cells at room temperature for 2 h, followed by incubation with 4,6-diamidino-2-phenylindole (DAPI) (Sigma-Aldrich, St. Louis, MO, USA) for 10 min at room temperature. A confocal laser scanning microscope and Zen2011 software was used to analyze the fluorescence.

### EdU incorporation assay

The incorporation of 5-ethynyl-2′-deoxyuridine (EdU) was measured using an EdU kit (RiboBio, Guangzhou, China) following the manufacturer’s protocol. Briefly, cells were exposed to DCZ3301 for 48 h and harvested. 2 h before collection, cells were treated with 50 μM EdU at 37 °C. Then cells were harvested and resuspended with PBS, followed by permeabilization with TritonX-100 (Sigma-Aldrich, St. Louis, MO, USA). Azide-conjugated Alexa Fluor 567 dye and Hoechst 33342 were used to dye the cells. At last we observed the DCZ3301-induced proliferation inhibition using fluorescence microscopy.

### Western blot analysis

Briefly, cells were collected and resuspended at 4 °C. Then we fractionated the proteins in 10% or 12.5% SDS-PAGE and transferred to nitrocellulose membrane. 5% skim milk was used to block the membranes at room temperature for 1 h. After that, those membranes were incubated with primary antibodies overnight at 4 °C. Fluorescence-conjugated secondary antibodies were added to incubated with membranes the other day. The expression was measured by Odyssey infrared imaging system (LI-COR Biosciences, Lincoln, USA). The β-actin was used to normalize the amount of protein in each sample.

The primary antibodies cleaved caspase 3 (#9661, 1:1000), cleaved caspase 8 (#9496, 1:1000), cleaved caspase 9 (#9532S, 1:1000), Bax (#2772, 1:1000), Bcl-2 (#2872, 1:1000), Cdc25C (#4688, 1:1000), phospho-Cdc25C (#32051, 1:1000), Histone H3 (#4499,1:1000), phospho-Histone H3 (#3377S, 1:1000), β-actin (#3700, 1:1000), Rad51 (#8875, 1:1000) were from Cell Signaling Technology (CST, Beverly, MA,USA). Cyclin B1 (#ab32052,1:1000), CDK1 (#ab32384,1:1000), γH2A.X (#ab2893, 1:1000), Histone H2A.X (#ab124781,1:1000), phospho-ATM (#ab81292, 1:1000), ATM (#ab32420,1:1000), phospho-ATR (#ab178407, 1:1000), phospho-CHK1 (#ab47318,1:1000), CHK1 (#ab40866, 1:1000), phospho-CHK2 (#ab85742, 1:1000), CHK2 (#ab47433, 1:1000), phospho-DNA PKCs (#ab124918, 1:1000) were from Abcam (Abcam, Cambridge, UK). BRCA1 (#sc135732, 1:200), BRCA2 (#sc293185, 1:200) were from Santa Cruz (Santa Cruz, California, USA).

### NHEJ and HR reporter assay

NHEJ and HR reporter assays were carried out according to a previously published report [16, 17]. Briefly, we use HEK-293 T cell line as the standard model cell line for this experiment. Lipofectamine 2000 transfection agent (Invitrogen, MA, USA) was used to transfect a mutant GFP plasmid containing a specific Isce-I site (DR-GFP) and an Isec-I-expressing plasmid (Isce-I) or NHEJ plasmid to HEK-293 T cells according to the instructions. After 5 h, the old culture medium was removed and new culture medium containing 8 μM DCZ3301 was added. After 24 h, the percentage of GFP positive cells (GFP%) was assessed using a FACS Canto II flow cytometer (BD Bioscience, California, USA) and the HR or NHEJ percentages were calculated using the formula:

$$ \left(\mathrm{Percentages}\ \mathrm{of}\ \mathrm{HR}\ \mathrm{or}\ \mathrm{NHEJ}\right)=\left(\mathrm{GFP}\%\mathrm{of}\ \mathrm{HR}\ \mathrm{infected}\ \mathrm{or}\ \mathrm{NHEJ}\ \mathrm{transfected}\ \mathrm{cells}\right)\div \left(\mathrm{GFP}\%\mathrm{of}\ \mathrm{corresponding}\ \mathrm{GFP}\ \mathrm{Vector}\ \mathrm{transfected}\ \mathrm{cells}\right) $$

### In vivo animal experiments

BALB/C nude mice were bought from the Shanghai Laboratory Animal Center (SLAC, Shanghai, China). Resuspend NCI-H929R cells with serum-free culture medium, mix the cell resuspension with the Matrigel (Corning, NY, USA) in proportion to 1:1 according to the instruction. Subcutaneously inject 100 μL mixture into the upper flank of nude mice rapidly. The density of cells in the mixture is about 4 × 10^8^ cells/mL. When the tumors were visible, mice were randomly divided into control group (vehicle, daily), BTZ group (1 day of 0.5 mg/kg BTZ followed by 2 days of vehicle), DCZ3301 group (1 day of vehicle followed by 2 days of 50 mg/kg DCZ3301) and combination group (1 day of 0.5 mg/kg BTZ followed by 2 days of 50 mg/kg DCZ3301, cyclical repetition). Mice were injected for 20 days. The tumor volume was calculated every two days and calculated as 4 × 3.14/3 × (width/2)^2^ × (length/2). Mice were sacrificed by cervical dislocation. Tumor specimens were fixed with 4% paraformaldehyde. We also performed hematoxylin and eosin (H&E), Ki-67, cleaved-caspase 3, TUNEL, γ-H2A.X, and phospho-CHK1 staining. The relative protein expressions were assessed by Image-pro plus. Version 6.0.0.260 (Media Cybernetics Inc., Maryland, USA).

In order to test the acute toxicity of DCZ3301 in vivo, we injected vehicle or 50 mg/kg DCZ3301 into healthy BALB/C nude mice intraperitoneally for 2 weeks. Then we analyzed the level of glutamic-pyruvic transaminase (ALT), glutamic-oxalacetic transaminase (AST), creatinine (Cr), and urea nitrogen (BUN) in serum. Alanine aminotransferase and aspartate aminotransferase assay kit (Nanjing Jiancheng Bioengineering Institute, China) were used for determining the level of ALT, AST, Cr and BUN. In this study, all animal-related experimental operations were licensed by the Animal Care and Use Committee of Shanghai Tenth People’s Hospital, Tongji University.

### Statistics

We performed all experiments in triplicate. The statistical significance was measured using the Student’s t-test or one-way variant analysis (ANOVA) with SPSS 20.0 (SPSS Inc., Chicago, IL, USA). Results with *p* < 0.05 were identified to be significant. Combination Index (CI) was used to evaluate synergistic and antagonistic interactions. And we use CalcuSyn software program (CalcuSyn; Biosoft, USA) to calculated the relative CI value.

## Results

### DCZ3301 treatment countered BTZ resistance and exhibited potent cytotoxicity against resistant MM cells

DCZ3301 is a novel aryl-guanidino compound whose molecular structure is shown in Fig. 1a. The BTZ-resistant MM cell lines NCI-H929R and RPMI-8226R5 were used in this study. The process of cell line establishment is presented in Fig. 1b. The established cell lines were treated with BTZ to confirm their resistance to the drug. Both cell lines were highly resistant to BTZ (NCI-H929R IC_50_: 48.7 ± 3.2 nM, RPMI-8226R5 IC_50_: 34.6 ± 2.3 nM) (Fig. 1c) after 48 h BTZ treatment compared to the BTZ-sensitive cell lines (NCI-H929S IC_50_: 15.6 ± 1.2 nM, RPMI-8226 IC_50_: 9.8 ± 1.1 nM). These results confirmed the BTZ resistance of the established cell lines. Next, we carried out the CCK8 assay and the soft agar clonogenic assay to investigate the anti-proliferative activity of DCZ3301 on BTZ-resistant MM cells. BTZ-resistant cell lines were treated with varying concentrations of DCZ3301 (2–32 μM) for 24, 48 and 72 h. DCZ3301 treatment decreased the cell viability in a time- and dose-dependent manner (Fig. 1d). Moreover, DCZ3301 treatment significantly inhibited the colony formation by both cell lines, indicating that DCZ3301 inhibited BTZ-resistant MM cell proliferation (Fig. 1e). EdU incorporation was significantly suppressed in DCZ3301 treated BTZ-resistant MM cells (Fig. 1f). These results demonstrated that DCZ3301 markedly affected the proliferation of BTZ-resistant MM cells.

### DCZ3301 induced apoptosis in BTZ-resistant MM cells and relapsed/refractory primary MM cells

**Fig. 2:**
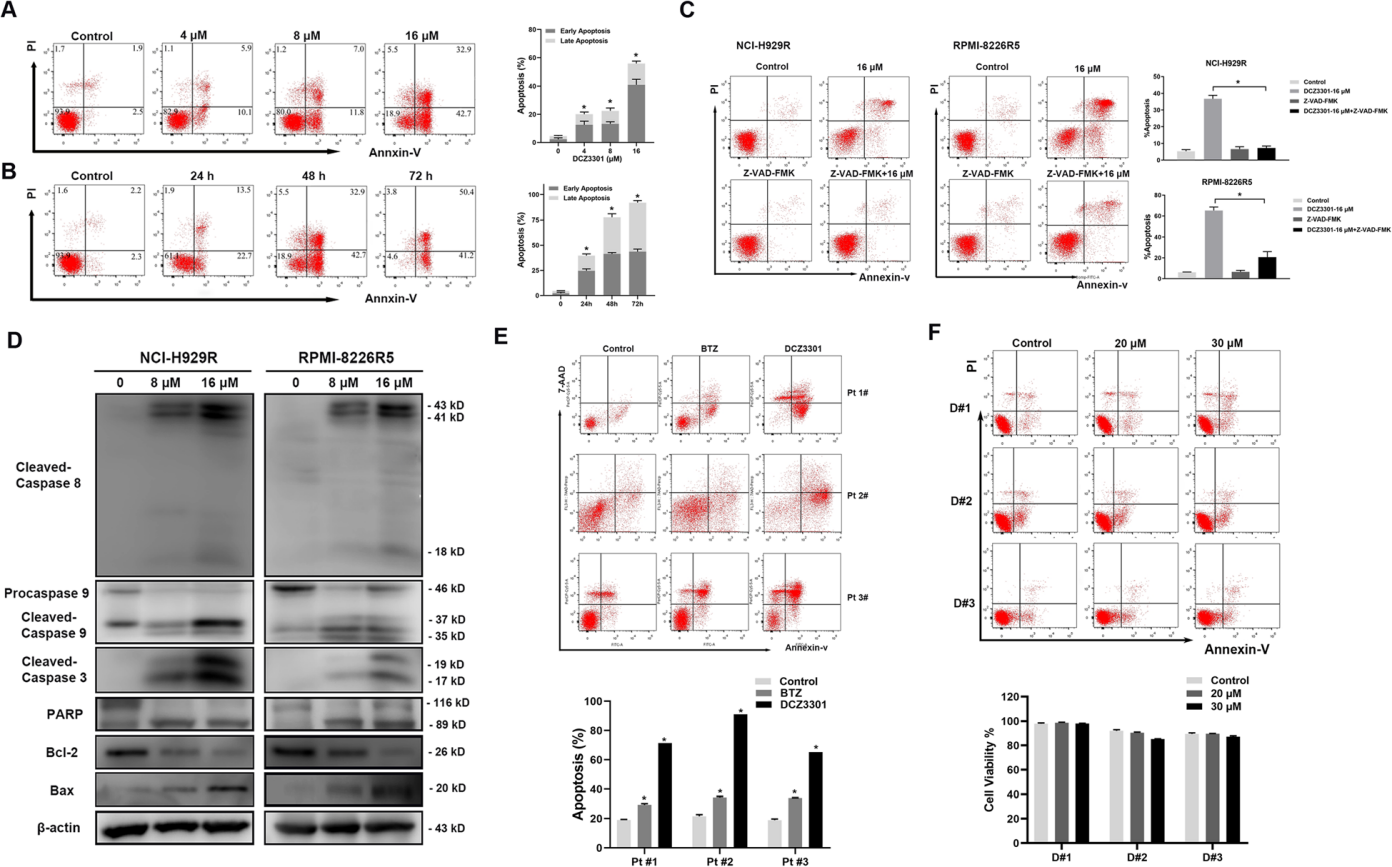
DCZ3301 treatment resulted in apoptosis in BTZ-resistant MM cells. (**a**) NCI-H929R were treated with DCZ3301 in the indicated concentrations (4 μM, 8 μM, 16 μM) for 48 h, and apoptosis was evaluated with Annexin V/PI staining and flow cytometry. The sum of early apoptotic cells (Annexin V^+^/PI^−^) and late apoptotic cells (Annexin V^+^/PI^+^) form total apoptosis. The percentage of apoptotic cells induced by increasing the concentration of DCZ3301 is shown on the right panel. (**b**) NCI-H929R treated with 16 μM DCZ3301 for the indicated time (24 h, 48 h, 72 h). Quantification of apoptotic cells is shown in the right panel. (**c**) DCZ3301 (16 μM) and/or the pan-caspase inhibitor Z-VAD-FMK (50 μM) were added for 48 h, and then we analyzed the percentage of apoptosis. (**d**) Western blot of the expression of apoptosis-related proteins. (**e**) Apoptosis in CD138^+^ primary MM cells isolated from three BTZ refractory and relapsed MM patients (Pt) treated with BTZ (40 nM) and DCZ3301 (16 μM) for 48 h. (**f**) Apoptosis in PBMCs from three healthy donors treated with DCZ3301 for 48 h

We performed flow cytometry and western blot to examine whether the anti-proliferative activity exhibited by DCZ3301 involved apoptosis induction in treated cells. Treatment of NCI-H929R cells with various concentrations (4 μM, 8 μM, 16 μM) of DCZ3301 for 48 h led to a significant increase in the number of early-stage (Annexin-V^+^/PI^−^) and the late-stage (Annexin-V^+^/PI^+^) apoptotic cells compared to the DMSO-treated control cells (Fig. 2a). Furthermore, the pro-apoptotic effect induced by 16 μM DCZ3301 treated for 24 h,48 h and 72 h increased in a time-dependent manner (Fig. 2b). We could inhibit the pro-apoptotic effect by Z-VAD-FMK, a pan-caspase inhibitor (Fig. 2c). Further, we performed western blot to evaluate the effects of DCZ3301 on the expression of caspase enzymes, and the anti-apoptotic Bcl-2 and pro-apoptotic Bax proteins. Our results showed that 48 h treatment of BTZ-resistant MM cells with DCZ3301 enhanced the cleavage of caspase-3, 8, and 9 and poly (ADP)-ribose polymerase (PARP) in a dose-dependent manner. Furthermore, DCZ3301 treatment decreased and increased the expression of Bcl-2 and Bax, respectively (Fig. 2d). To validate these results in patient-derived cells, (CD138^+^) BTZ-resistant MM cells from three refractory/relapsed MM patients were treated with DC3301. DCZ3301 significantly induced apoptosis in the primary MM cells (Fig. 2e). In contrast, DCZ3301 treatment at concentrations as high as 30 μM failed to induce significant cytotoxicity in PBMCs from three healthy volunteers (Fig. 2f).

### DCZ3301 induced M phase arrest in BTZ-resistant MM cells

**Fig. 3:**
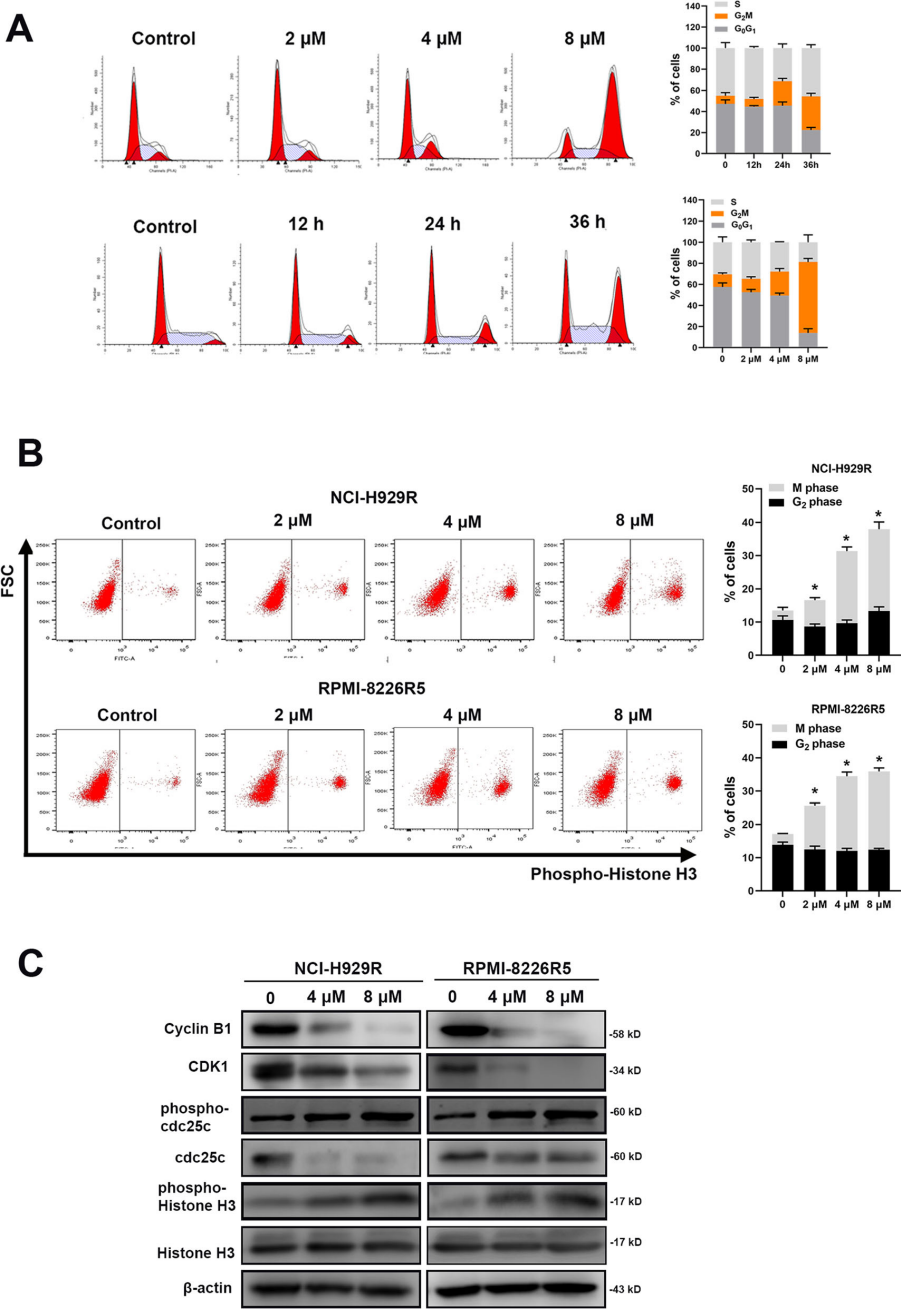
DCZ3301 caused M phase arrest in BTZ-resistant cells. (**a**) NCI-H929R treated with DCZ3301 with indicated concentrations (the upper panel) and for the indicated time (lower panel) and cell cycle arrest analyzed using PI staining and flow cytometry. (**b**) The percentage of M phase arrest induced by DCZ3301 was tested by staining with Alexa Fluor®488-conjugated anti-phospho (Ser10)-Histone H3 polyclonal antibody and flow cytometry. Quantification of M phase cell arrest induced by DCZ3301 is shown on the right panel. Data represent the mean of three independent experiments. *p < 0.05 (**c**) Western blot showing the expression of G_2_/M phase-related proteins in BTZ-resistant MM cells treated with DCZ3301

In addition to apoptosis induction, the inhibition of the cell cycle progression may be an important efficacy parameter of anti-cancer drugs. Therefore, we evaluated the effects of DCZ3301 on cell cycle progression. DCZ3301 treatment induced a significant accumulation of cells in G_2_/M in a dose- and time-dependent manner (Fig. 3a). To further elucidate the mechanisms underlying DCZ3301 induction of G_2_/M phase arrest, we analyzed the DCZ3301-mediated cell cycle arrest of BTZ-resistant cells using an anti-phosphorylated Histone H3 antibody. Phosphorylation at Ser10 of Histone H3 is strongly correlated with chromosome condensation during mitosis, and the M phase can be identified using the anti-phosphorylated Histone H3 antibody [18]. Interestingly, results from both flow cytometry and western blot analyses confirmed that treatment with DCZ3301 upregulated the level of phosphorylated Histone H3 (Fig. 3b-c), indicating that DCZ3301 treatment specifically increased the M phase cell cycle arrest without influencing the number of the G_2_ phase cells. Simultaneously, the expression of several G_2_/M phase proteins was modulated by DCZ3301 (Fig. 3c). DCZ3301 reduced cyclin B1, CDK1, and Cdc25C expression and upregulated the levels of phosphorylated Cdc25C.

### DCZ3301 enhanced the DNA damage response and impaired DNA repair in BTZ-resistant cells

**Fig. 4:**
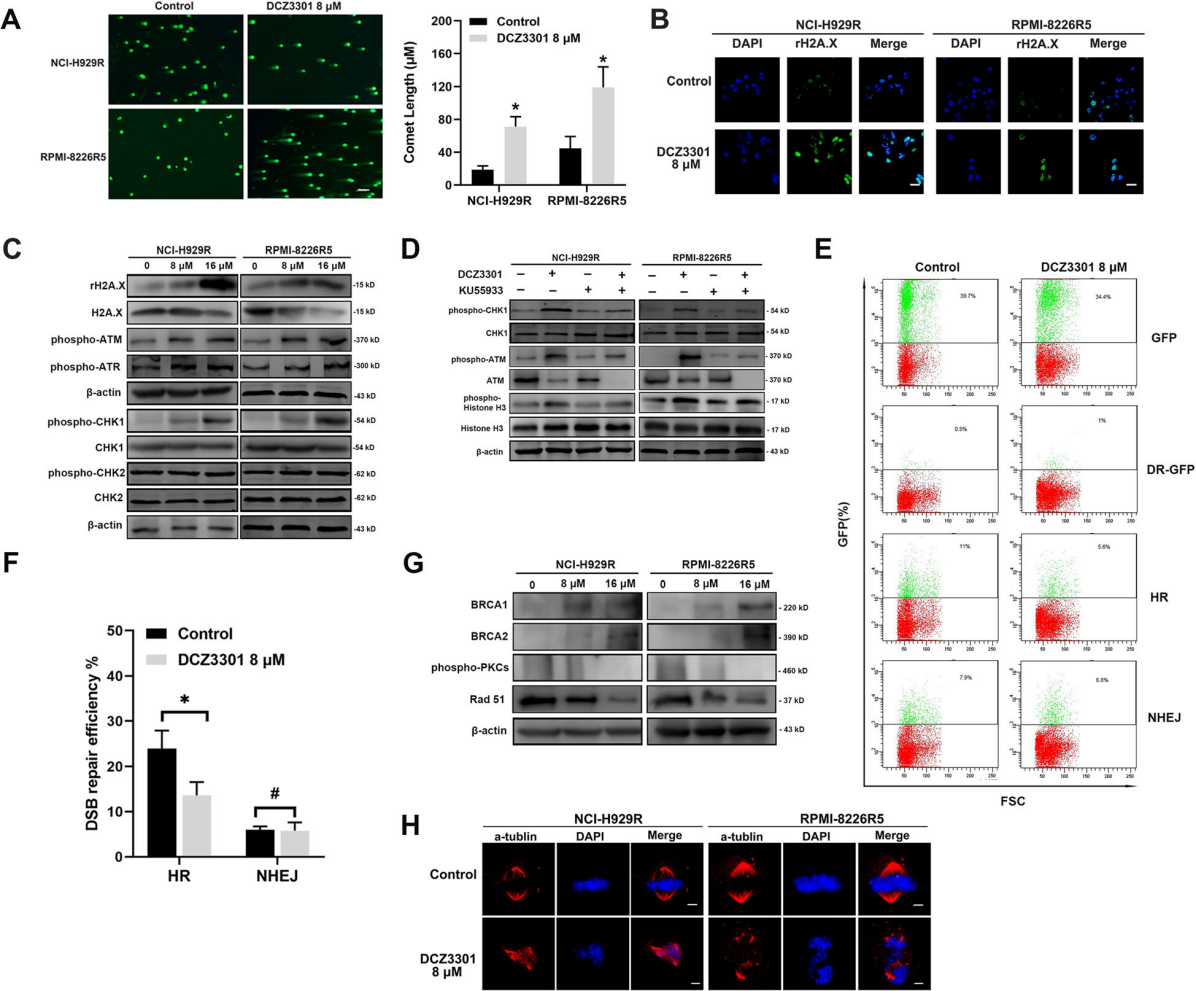
DCZ3301 induced mitotic catastrophe. (**a**) After treatment with DCZ3301 for 48 h, the comet assay was carried out to evaluate the degree of DNA damage. Scale bars = 100 μm. (**b**) Immunofluorescence staining of γ-H2A.X in BTZ-resistant NCI-H929R and RPMI-8226R5 cells after treatment with DCZ3301 (8 μM) for 48 h. Scale bars = 5 μm. (**c**) DCZ3301 activated the ATM-ATR/CHK1 signaling pathway. (**d**) Western blot analysis showing DCZ3301-mediated activation of the ATM/CHK1 signaling pathway. (**e**) HR and NHEJ reporter assays of HR repair efficiency and NHEJ repair efficiency in HEK-293 T cells treated with DCZ3301. (**f**) Quantification of HR and NHEJ repair efficiency. Data are presented as mean ± SD. *p < 0.05, #*p* > 0.05. (**g**) Expression of DNA repair related proteins in NCI-H929R and RPMI-8226R5 treated with DCZ3301 for 48 h. (**h**) Mitotic catastrophe induced by DCZ3301. DNA was stained with DAPI (blue) and the spindles were stained with α-tubulin (red). Scale bars = 5 μm

The M phase arrest is often related to DNA damage, and DCZ3301 specifically induced the M phase arrest in BTZ-resistant MM cells. We treated NCI-H929R and RPMI-8226R5 cells with DCZ3301 for 48 h and subsequently performed the comet assay to measure the double-strand breaks (DSBs) in DNA. The characteristic comet tails were observed in DCZ3301-treated cells, suggesting that the compound enhanced DNA damage in BTZ-resistant cells (Fig. 4a). The level of γ-H2A.X has been used as a marker to detect and quantify the early phase of DSBs [19]. In this study, we used immunofluorescence (IF) analysis to evaluate the γ-H2A.X levels, and our results revealed a higher γ-H2A.X expression in DCZ3301 treated cells than baseline γ-H2A.X levels (Fig. 4b). These results were supported by parallel DCZ3301-mediated upregulation of γ-H2A.X, phospho-ATM, and phospho-ATR, the key proteins involved in DNA DSBs (Fig. 4c). Moreover, we checked the expression of downstream proteins, and found that the phosphorylation of CHK1 were activated (Fig. 4c). However, the level of phosphorylated CHK2 was not affected. Based on the fact that DCZ3301 was an anti-cancer agent that induced DNA damage and the M phase cell cycle arrest, we speculated that DCZ3301 might also activate the ATM-ATR/CHK1 signaling pathway. Towards this, both NCI-H929R and RPMI-8226R5 cells were treated with DCZ3301 and subsequently exposed to KU55933, an ATM inhibitor. Our western blot results revealed that KU55933 could reverse the DCZ3301-mediated upregulation of phospho-CHK1 and phospho-ATM (Fig. 4d). Furthermore, KU55933 treatment modulated the phosphorylation of Histone H3, indicating that the M phase cell cycle arrest could be attributed to the activation of the ATM/CHK1 signaling pathway.

A DNA damage response for the efficient and appropriate repair of DSBs is essential for the preservation of genomic integrity [19]. However, the repair of genotoxic agent-induced DSBs by the non-homologous end joining (NHEJ) or HR repair pathways promotes resistance and subsequent relapse in patients [20]. Thus, we used GFP-based repair assays to evaluate whether DCZ3301 could impair the DNA repair pathways. Our results showed that DCZ3301 suppressed HR but had little effect on NHEJ (Fig. 4e and f). Moreover, western blot showed that DCZ3301 could activate BRCA1/2 and decrease the expression of phospho-DNA PKCs and RAD51, the key regulators of DNA repair (Fig. 4g). DNA damage and aberrant DNA repair usually turn cells into aneuploids, which may lead to mitotic catastrophe [21]. Our results showed that following treatment with DCZ3301, both NCI-H929R and RPMI-8226R5 cells exhibited multipolar mitotic spindles (Fig. 4h).

### DCZ3301 exhibited synergistically with BTZ in vitro

**Fig. 5:**
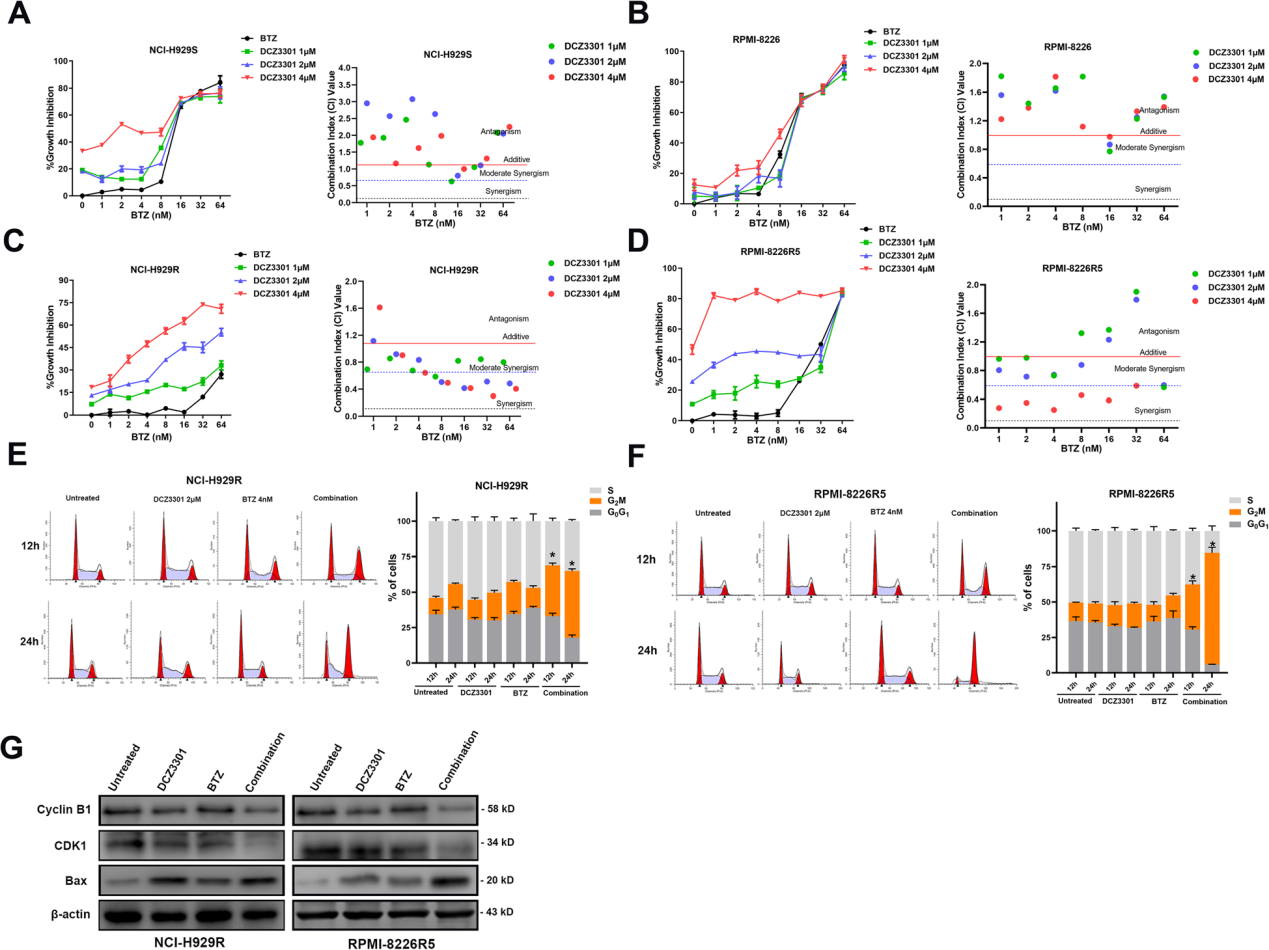
Synergistic effect of DCZ3301 and BTZ in MM cell lines. Effect of treatment with BTZ alone- or in combination with different doses of DCZ3301 on cell growth in NCI-H929S (**a**), RPMI-8226 (**b**), NCI-H929R (**c**), and RPMI-8226R5 (**d**) cells. Combination index (CI) for two drugs in the MM cell lines. CI value < 1 represents synergistic. Data were expressed as mean ± SD. (**e** and **f**) Cell cycle analysis of BTZ-resistant MM cells treated with DCZ3301 alone, BTZ alone, and in combination with DCZ3301. (**g**) Western blot of G_2_/M phase related proteins in NCI-H929R and RPMI-8226R5 cells treated with DCZ3301 alone, BTZ alone and their combination for 48 h

Since DCZ3301 had a cytotoxic effect on BTZ-resistant MM cells, we questioned whether DCZ3301 treatment could re-sensitize BTZ-resistant MM cells. To answer this, we added both DCZ3301 and BTZ simultaneously to the MM cell culture and calculated the combination Index (CI) between DCZ3301 and BTZ. The CI is considered the gold standard to define the synergism of drug-drug interactions [22, 23]. CI values =1 always represents an additive effect, while CI values < 1 and > 1 indicate synergistic and antagonistic interactions, respectively. Thus, a low CI value stands for strong synergism and vice versa. According to our previous study, DCZ3301 showed synergism with BTZ in MM.1S (BTZ-sensitive MM cells) [15]. In the current study, we observed similar results in the BTZ-sensitive MM cells, NCI-H929S, and RPMI-8226 (Fig. 5a and b). Interestingly, DCZ3301 showed a higher synergistic effect with BTZ in the BTZ-resistant cell lines NCI-H929R and RPMI-8226R5 than in BTZ-sensitive MM cells (Fig. 5c and d). The detailed concentrations of DCZ3301 and BTZ are presented in Supplementary Table 1–8. Furthermore, DCZ3301 was synergistic with the histone deacetylase (HDAC) inhibitor panobinostat in NCI-H929R and RPMI-8226R5 cells (Supplementary Figure 1) over a broad range of concentrations, and the synergy increased with higher panobinostat doses. To understand the underlying mechanism behind the efficacy of combination treatment, cell cycle analysis was carried out for BTZ-resistant cells via flow cytometry. More BTZ-resistant cells were observed in G_2_/M phase arrest when treated with combination therapy than when treated with a single compound (Fig. 5e and f). In addition, the expression of cyclin B1 and CDK1 was further decreased, whereas the expression of the apoptosis marker Bax was higher in cells subjected to combination treatment (Fig. 5g).

### Synergistic effect of DCZ3301 and BTZ against BTZ-resistant MM in vivo

**Fig. 6:**
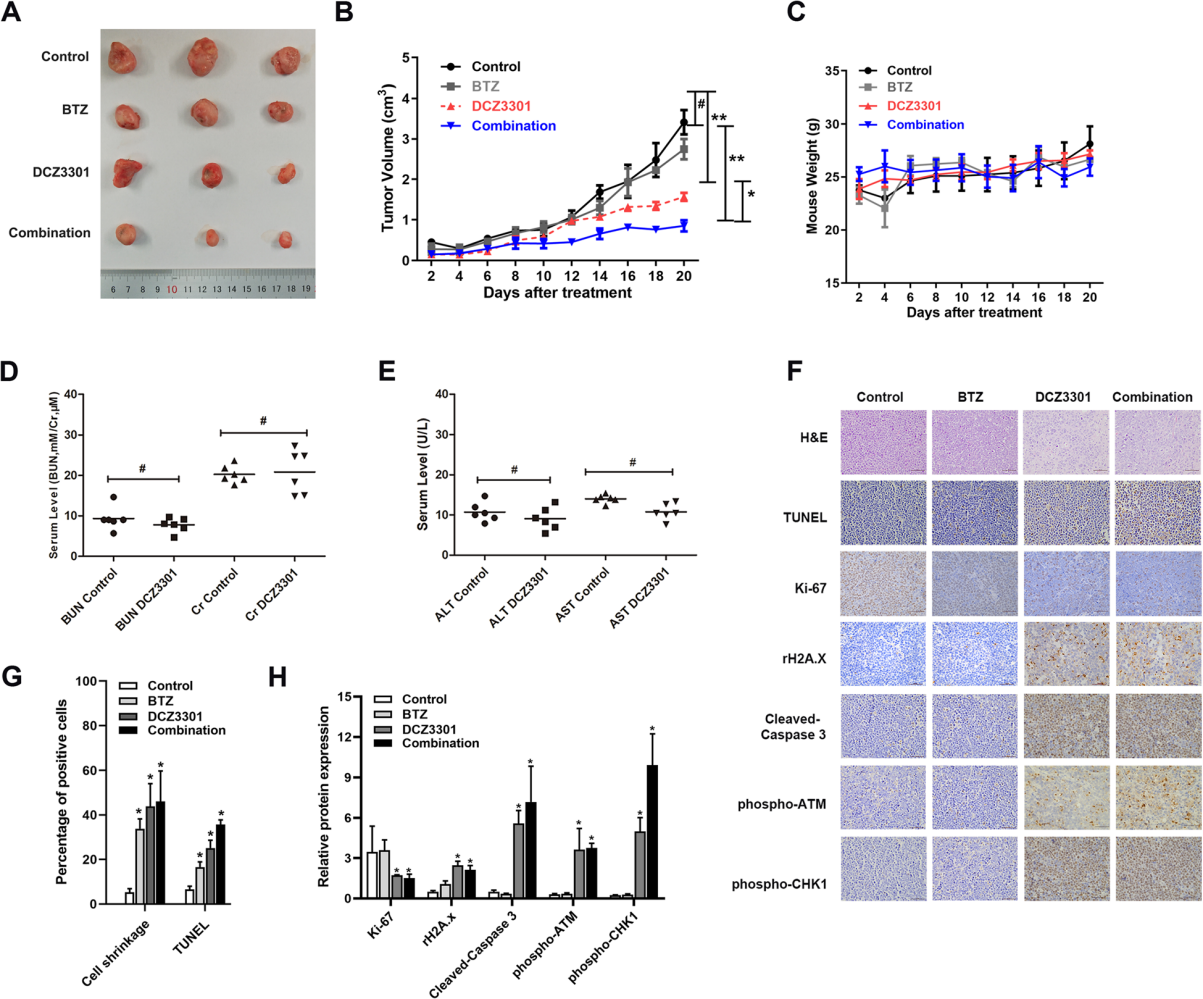
Synergistic effect of DCZ3301 and BTZ against BTZ-resistant MM in vivo*.* (**a**) Gross appearance of tumors on day 20. (**b**) Tumor growth curves of 20 days treatment. (**c**) Growth curve of mouse weight (*n* = 3 for each group). (**d**) and (**e**) Serum levels of ALT, AST, Cr and BUN (*n* = 6 for each group). *p < 0.05, #*p* > 0.05 Data were represented as mean ± SD. (**f**) H&E staining of tumor sections for tumor histology after treatment. TUNEL, Ki-67, γ-H2A.X, cleaved caspase-3, phospho-ATM and phospho-CHK1 were stained immunohistochemically in tumor sections. (**g**) The percentage of cell shrinkage and TUNEL-positive cells in tumor sections. (**h**) The relative protein expressions of Ki-67, γ-H2A.X, cleaved caspase-3, phospho-ATM and phospho-CHK1 quantified by Image Pro-plus in tumor sections

We validated these in vitro findings in in vivo experiments. Combinational treatment induced greater tumor inhibition than treatment with BTZ or DCZ3301 alone (Fig. 6a and b), indicating that the treatment of BTZ-resistant cells with DCZ3301 could restore the sensitivity to BTZ in vivo. The body weight of the mice was monitored during the treatment to evaluate potential side effects, and 20-day treatment with DCZ3301 did not cause any changes in the animal weight (Fig. 6c). Besides, no significant differences in the serum levels of ALT, AST, Cr, or BUN were observed in mice treated with DCZ3301 (50 mg/kg) (Fig. 6d and e), indicating that it was well tolerated. H&E staining showed that cell shrinkage and fragmentation increased upon DCZ3301 and combinational treatment (Fig. 6f). Further, we carried out the TUNEL staining to detect apoptosis and immunohistochemical staining (IHC) to measure the expressions of Ki-67, γ-H2A.X, cleaved-caspase 3, phospho-ATM and phospho-CHK1 (Fig. 6f). And the percentages of cell shrinkage and TUNEL-positive cells were quantified with image-Pro plus. Both the percentage of cell shrinkage and TUNEL-positive cells were increased in DCZ3301 and combination treatment groups (Fig. 6g). The results of quantified relative protein expressions in Fig. 6h showed that Ki-67 expression was downregulated, whereas the cleavage caspase-3 were upregulated by both DCZ3301 alone and combinational treatment. Moreover, consistent with our in vitro results, the expression of γ-H2A.X, phospho-ATM, and phospho-CHK1 were upregulated in vivo. Collectively, the data of the present study suggest that DCZ3301 is a promising compound to counter BTZ resistance in MM in vivo and in vitro.

## Discussion

Acquired drug resistance can be the result of the activation of an alternative compensatory signaling pathway [21], mutations or quantitative alterations that arise during therapy, or various adaptive responses. In this study, we established two BTZ-resistant cell lines by increasing the concentration of BTZ in a step-wise manner. DCZ3301 inhibited cell proliferation in a dose- and time-dependent manner. The flow cytometric results confirmed that DCZ3301-mediated pro-apoptotic effects were specific to the BTZ-resistant cells, since no significant apoptosis was detected in PBMCs treated with up to 30 μM DCZ3301. Both the G_2_ and M phase belong to the late stage of mitosis, and cells in these phases have the same DNA content. However, one of the most remarkable differences between the G_2_ and M phase is the chromatin condensation in the G_2_ phase and chromosome formation in the M phase. The phosphorylation of Histone H3 Ser 10 is correlated with the progression of chromatin condensation [18, 24]. We found that after DCZ3301 treatment the phosphorylation of Histone H3 was significantly upregulated. This indicated that DCZ3301 inhibited BTZ-resistant cells in the M phase and not the G_2_ phase.

Next, we investigated the influence of DCZ3301 on the expression of G_2_/M checkpoint proteins. The checkpoint pathways involved in DNA damage or errors are phylogenetically conserved according to the previous report. The function of active checkpoints is delaying cell cycle progression to facilitate DNA repair [21]. CHK1 and CHK2 are major effectors of cell cycle regulation in these checkpoint proteins [25, 26]. During DNA damage, the key regulators in the checkpoint pathways, ATM and ATR kinases, are activated by phosphorylation that in turn phosphorylates H2A.X via the checkpoint kinases CHK1 or CHK2 to induce cell cycle arrest [12]. During the G_2_ phase, CHK1 phosphorylates and suppresses Cdc25-A, −B, and -C, thereby preventing cyclin B1/cdk1 activation, eventually leading to G_2_ arrest [27, 28]. Thus, the G_2_ checkpoint is the last opportunity to halt the cycle and repair the DNA damage in cells that have escaped the G_1_ and S phase checkpoints.

Based on this information, we investigated whether DCZ3301 activated any of the upstream DNA damage signaling pathways. The comet assay and IF results showed that DCZ3301 aggravated DNA fragmentation. Also, the expression of phosphorylated CHK1 was upregulated. The protein γ-H2A.X Ser 139 is regarded as a surrogate marker for DNA double-strand breaks [19, 29]. Activation of the DNA damage response includes the formation of DNA damage foci containing activated γ-H2A.X. Therefore, we examined the expression of γ-H2A.X and formation of γ-H2A.X foci using western blot and IF. DCZ3301 treatment upregulated γ-H2A.X expression and promoted the formation of γ-H2A.X foci. Further, DCZ3301 upregulated the phosphorylation of ATR, ATM, and their downstream proteins CHK1, and these effects could be blocked by KU55933, an ATM specific inhibitor. Collectively, the results confirmed that DCZ3301 induced DNA damage in BTZ-resistant MM cells.

We contemplated why DCZ3301-mediated DNA damage blocked BTZ-resistant MM cells in the M phase, and not the G_2_ phase. The CyclinB1-CDK1 complex plays a regulatory role during the G_2_/M phase [30]. During DNA damage, CHK1 and CHK2 kinases phosphorylate and inactivate Cdc25C [31], which in turn activates CDK1 by dephosphorylating it. Therefore, the G_2_ checkpoint is the last opportunity to halt the cycle and repair DNA damage in cells that have escaped the G_1_ and S phase checkpoints [32]. During the G_2_ phase, HR is the predominant pathway responsible for DNA repair [33]. Our results showed that DCZ3301 treatment impaired HR activity without affecting NHEJ activity in treated cells, thereby leading to irreparable DNA damage. This was associated with the downregulation of Rad51 [34]. However, in our study BRCA1 and BRCA2 were overexpressed. This response may be part of a DNA repair mechanism that lead to an initial upregulation of BRCA1 and BRCA2 in response to DCZ3301-mediated DNA damage. However, subsequent repair might be suppressed for some unknown reason. When DNA damage is not detected or repaired by the end of the G_2_ phase, it leads to the activation of the cyclin B1-CDK1 complex by Cdc25 phosphatase [35]. In our study, DCZ3301 treatment decreased the expression of cyclin B1, CDK1, and Cdc25C, whereas it increased the phosphorylation of Cdc25C. Interestingly, flow cytometry showed that BTZ-resistant cells were blocked in the M phase and not the G_2_ phase, which indicated that these cells might have “slipped” in the M phase with damaged DNA. We observed Histone H3 phosphorylation, which indicated that chromatin was indeed condensed during the M phase. The premature mitotic entry of defective cells before proper chromosome segregation leads to mitotic catastrophe and cell death upon entering mitosis [36]. Our IF results showed that DCZ3301 treatment led to a mitotic catastrophe and confirmed these speculations.

Mitotic catastrophe shares several biochemical hallmarks of apoptosis [37]. We observed caspase-dependent apoptosis induced by DCZ3301 in vitro and necrosis in xenotransplanted tumors in vivo. Significantly, unlike normal cells, most cancer cells have aberrant cell cycle checkpoints, especially G_2_ phase checkpoint, and are prone to mitotic catastrophe [21]. This explains the specificity of DCZ3301 in inducing the mitotic catastrophe in cancer cells but not in PBMCs. BTZ is reported to induce mitotic catastrophe through the ubiquitin-proteasome system in B-cell lymphoma cells [38]. However, BTZ resistance can arise through the therapy-induced selection of a minor resistant cell subpopulation present in MM [21]. Based on our results, we propose that DCZ3301 acts synergistically with BTZ and reprograms BTZ-resistant cells to restore their sensitivity through complementary mechanisms.

## Conclusions

**Fig. 7:**
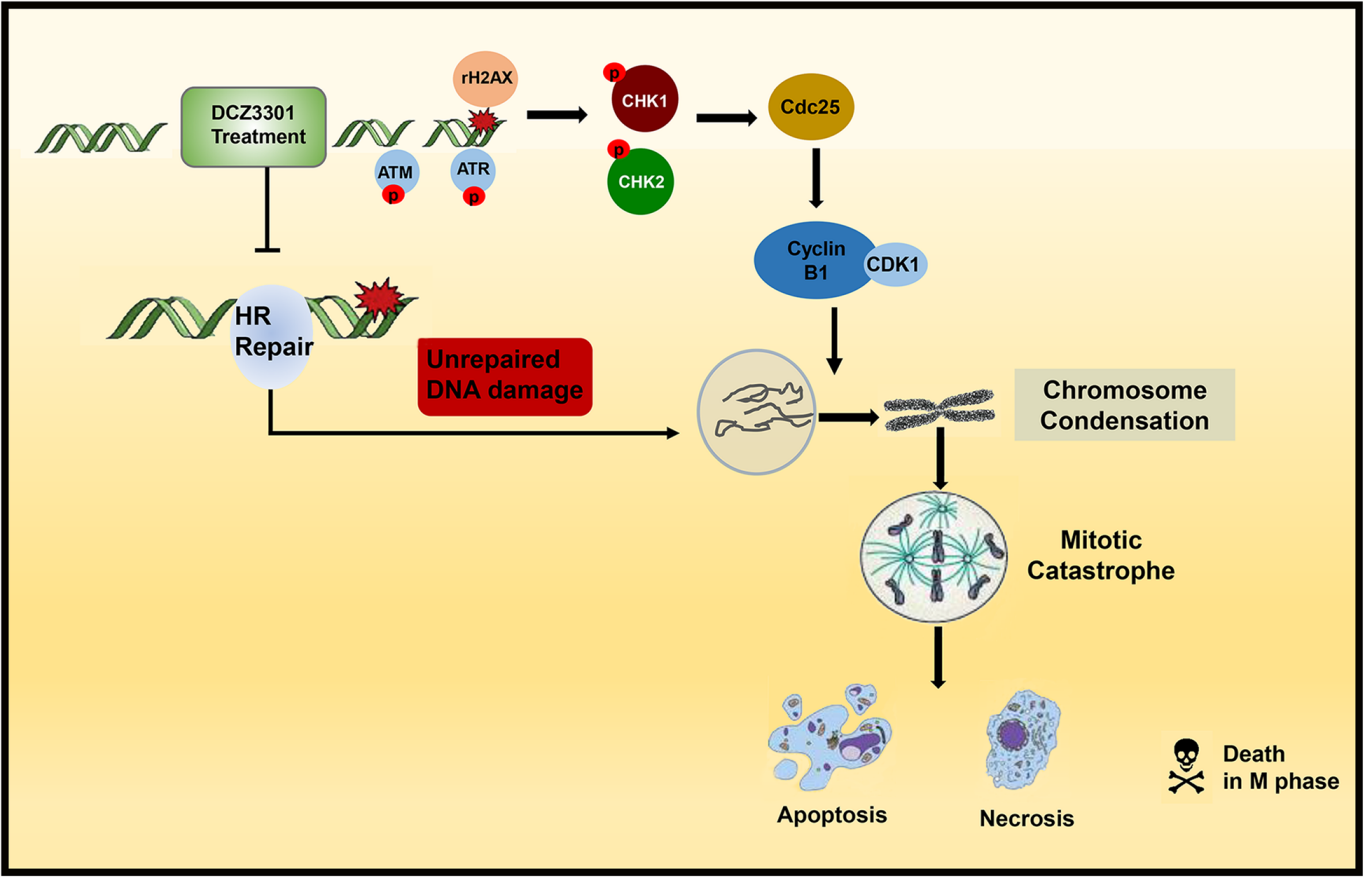
Diagram illustrating the mechanism of DCZ3301 effect on DNA damage and G_2_/M checkpoint regulation

In conclusion, we identified the mechanism of action of DCZ3301, a novel compound previously reported by us, that can enhance the sensitivity of BTZ in resistant MM. It activates DNA damage, inhibits DNA repair, and induces mitotic catastrophe through the ATM-ATR/CHK/Cdc25C pathway (Fig. 7) in BTZ-resistant cells. In addition, DCZ3301 acts synergistically with BTZ in vitro and in vivo. It enhances the BTZ-induced G_2_/M phase arrest and leads cells into mitotic catastrophe through complementary mechanisms. Collectively, these findings suggest that DCZ3301 may hold promise for BTZ-resistant MM therapy and may provide an effective strategy for BTZ resistance prevention and treatment.

## Supplementary information

**Additional file 1: Supplementary Table 1–8.** Detailed concentrations of DCZ3301 and BTZ used in the synergistic experiments. **Supplementary Figure 1.** DCZ3301 was synergistic with the histone deacetylase (HDAC) inhibitor panobinostat in NCI-H929R and RPMI-8226R5 cells.

## Data Availability

The datasets supporting the conclusions of this article are included in this published article (and its supplementary information files).

## Abbreviations

ATM: Ataxia telangiectasia mutated
BTZ: Bortezomib
CHK1: Checkpoint kinase 1
CHK2: Checkpoint kinase 2
DDR: DNA damage response
DLBCL: Diffuse large B-cell lymphoma
DSBs: Double strand breaks
FDA: Food and Drug Administration
H&E: Hematoxylin & eosin
MM: Multiple myeloma
NHEJ: Non-homologous end joining
PARP: Poly (ADP)-ribose polymerase
SD: Standard deviation
