# Supplementary material for: A novel M phase blocker, DCZ3301 enhances the sensitivity of bortezomib in resistant multiple myeloma through DNA damage and mitotic catastrophe
Source: J Exp Clin Cancer Res. 2020 Jun 9;39:105. doi: 10.1186/s13046-020-01597-9 (PMC7285565; doi:10.1186/s13046-020-01597-9)
Supplement: Supplementary file 1 — Additional file 1: Supplementary Table 1–8. Detailed concentrations of DCZ3301 and BTZ used in the synergistic experiments. Supplementary Figure 1. DCZ3301 was synergistic with the histone deacetylase (HDAC) inhibitor panobinostat in NCI-H929R and RPMI-8226R5 cells. [file 13046_2020_1597_MOESM1_ESM.docx]

**Supplementary Table 1**

Cytotoxicity of Bortezomib Combination treatment with DCZ3301 in Bortezomib resistant cell line **NCI-H929R**.

|  | **Inhibition (%)** |  |  |  |  |  |  |  |
| --- | --- | --- | --- | --- | --- | --- | --- | --- |
|  | **Bortezomib 0 nM** | **Bortezomib 1 nM** | **Bortezomib 2 nM** | **Bortezomib 4 nM** | **Bortezomib 8 nM** | **Bortezomib 16 nM** | **Bortezomib 32 nM** | **Bortezomib 64 nM** |
| **DCZ3301 0 μM** | 0.00 | 1.72% ± 0.84% | 2.56% ± 1.13% | 0.32% ± 0.18% | 4.54% ± 0.75% | 2.05% ± 0.35% | 12.07% ± 0.39% | 27.14% ± 2.31% |
| **DCZ3301 1 μM** | 7.36% ± 0.71% | 13.73% ± 0.62% | 11.45% ± 0.64% | 15.52% ± 1.02% | 19.90% ± 0.51% | 17.29% ± 0.11% | 22.32% ± 2.00% | 33.11% ± 2.44% |
| **DCZ3301 2 μM** | 13.11% ± 1.15% | 16.84% ± 1.91% | 20.63% ± 0.98% | 23.23% ± 0.84% | 37.00% ± 1.15% | 45.78% ± 1.96% | 45.06% ± 2.88% | 55.32% ± 2.09% |
| **DCZ3301 4 μM** | 18.44% ± 0.29% | 22.76% ± 3.17% | 37.16% ± 2.10% | 47.48% ± 1.58% | 56.16% ± 1.72% | 62.79% ± 1.80% | 73.73% ± 0.33% | 70.93% ± 2.47% |

**Supplementary Table 2**

Combination Index of Bortezomib Combination treatment with DCZ3301 in Bortezomib resistant cell line **NCI-H929R**.

|  | **Combination Index** |  |  |  |  |  |  |
| --- | --- | --- | --- | --- | --- | --- | --- |
|  | **Bortezomib 1 nM** | **Bortezomib 2 nM** | **Bortezomib 4 nM** | **Bortezomib 8 nM** | **Bortezomib 16 nM** | **Bortezomib 32 nM** | **Bortezomib 64 nM** |
| **DCZ3301 1 μM** | 0.69 | 0.85 | 0.68 | 0.59 | 0.82 | 0.84 | 0.80 |
| **DCZ3301 2 μM** | 1.12 | 0.92 | 0.83 | 0.51 | 0.41 | 0.51 | 0.48 |
| **DCZ3301 4 μM** | 1.61 | 0.90 | 0.64 | 0.49 | 0.41 | 0.30 | 0.41 |

**Supplementary Table 3**

Cytotoxicity of Bortezomib Combination treatment with DCZ3301 in Bortezomib resistant cell line **NCI-H929S**.

|  | **Inhibition (%)** |  |  |  |  |  |  |  |
| --- | --- | --- | --- | --- | --- | --- | --- | --- |
|  | **Bortezomib 0 nM** | **Bortezomib 1 nM** | **Bortezomib 2 nM** | **Bortezomib 4 nM** | **Bortezomib 8 nM** | **Bortezomib 16 nM** | **Bortezomib 32 nM** | **Bortezomib 64 nM** |
| **DCZ3301 0 μM** | 0.00 | 2.78% ± 1.17% | 4.87% ± 0.39% | 4.37% ± 1.08% | 10.50% ± 0.67% | 67.14% ± 1.76% | 77.82% ± 0.96% | 84.15% ± 3.90% |
| **DCZ3301 1 μM** | 19.10% ± 0.60% | 13.93% ± 0.68% | 12.38% ± 0.98% | 12.39% ± 0.09% | 35.61% ± 0.37% | 68.85% ± 0.98% | 73.48% ± 1.77% | 73.76% ± 3.92% |
| **DCZ3301 2 μM** | 18.27% ± 0.24% | 12.31% ± 1.90% | 19.99% ± 1.74% | 19.29% ± 1.86% | 24.14% ± 0.67% | 68.86% ± 0.30% | 74.90% ± 1.00% | 76.57% ± 3.64% |
| **DCZ3301 4 μM** | 33.27% ± 0.42% | 37.60% ± 0.95% | 53.18% ± 0.52% | 46.64% ± 0.28% | 47.31% ± 2.29% | 72.39% ± 0.64% | 75.86% ± 1.45% | 76.04% ± 2.39% |

**Supplementary Table 4**

Combination Index of Bortezomib Combination treatment with DCZ3301 in Bortezomib resistant cell line **NCI-H929S**.

|  | **Combination Index** |  |  |  |  |  |  |
| --- | --- | --- | --- | --- | --- | --- | --- |
|  | **Bortezomib 1 nM** | **Bortezomib 2 nM** | **Bortezomib 4 nM** | **Bortezomib 8 nM** | **Bortezomib 16 nM** | **Bortezomib 32 nM** | **Bortezomib 64 nM** |
| **DCZ3301 1 μM** | 1.78 | 1.93 | 2.46 | 1.13 | 0.63 | 1.05 | 2.08 |
| **DCZ3301 2 μM** | 2.95 | 2.57 | 3.08 | 2.63 | 0.80 | 1.11 | 2.05 |
| **DCZ3301 4 μM** | 1.94 | 1.17 | 1.62 | 1.98 | 1.00 | 1.31 | 2.25 |

**Supplementary Table 5**

Cytotoxicity of Bortezomib Combination treatment with DCZ3301 in Bortezomib resistant cell line **RPMI-8226R5**.

|  |  | **Inhibition (%)** |  |  |  |  |  |  |
| --- | --- | --- | --- | --- | --- | --- | --- | --- |
|  | **Bortezomib 0 nM** | **Bortezomib 1 nM** | **Bortezomib 2 nM** | **Bortezomib 4 nM** | **Bortezomib 8 nM** | **Bortezomib 16 nM** | **Bortezomib 32 nM** | **Bortezomib 64 nM** |
| **DCZ3301 0 μM** | 0.00 | 4.29% ± 0.27% | 3.84% ± 2.08% | 3.11% ± 1.50% | 5.08% ± 1.73% | 25.91% ± 0.56% | 50.04% ± 0.62% | 82.19% ± 0.66% |
| **DCZ3301 1 μM** | 10.94% ± 0.48% | 17.16% ± 2.02% | 17.85% ± 3.50% | 25.53% ± 3.33% | 23.84% ± 1.77% | 27.49% ± 0.48% | 34.84% ± 2.75% | 83.38% ± 1.69% |
| **DCZ3301 2 μM** | 25.65% ± 0.98% | 36.36% ± 1.46% | 43.85% ± 0.71% | 45.46% ± 0.76% | 44.81% ± 1.03% | 42.37% ± 0.76% | 43.45% ± 3.97% | 82.32% ± 1.17% |
| **DCZ3301 4 μM** | 46.56% ± 2.52% | 82.07% ± 1.43% | 78.95% ± 0.93% | 84.69% ± 1.48% | 78.25% ± 0.75% | 83.79% ± 0.23% | 81.48% ± 0.62% | 85.27% ± 1.37% |

**Supplementary Table 6**

Combination Index of Bortezomib Combination treatment with DCZ3301 in Bortezomib resistant cell line **RPMI-8226R5**.

|  | **Combination Index** |  |  |  |  |  |  |
| --- | --- | --- | --- | --- | --- | --- | --- |
|  | **Bortezomib 1 nM** | **Bortezomib 2 nM** | **Bortezomib 4 nM** | **Bortezomib 8 nM** | **Bortezomib 16 nM** | **Bortezomib 32 nM** | **Bortezomib 64 nM** |
| **DCZ3301 1 μM** | 0.96 | 0.98 | 0.73 | 1.32 | 1.37 | 1.90 | 0.57 |
| **DCZ3301 2 μM** | 0.81 | 0.71 | 0.74 | 0.88 | 1.23 | 1.79 | 0.60 |
| **DCZ3301 4 μM** | 0.28 | 0.35 | 0.25 | 0.46 | 0.38 | 0.59 | 0.59 |

**Supplementary Table 7**

Cytotoxicity of Bortezomib Combination treatment with DCZ3301 in Bortezomib resistant cell line **RPMI-8226**.

|  | **Inhibition (%)** |  |  |  |  |  |  |  |
| --- | --- | --- | --- | --- | --- | --- | --- | --- |
|  | **Bortezomib 0 nM** | **Bortezomib 1 nM** | **Bortezomib 2 nM** | **Bortezomib 4 nM** | **Bortezomib 8 nM** | **Bortezomib 16 nM** | **Bortezomib 32 nM** | **Bortezomib 64 nM** |
| **DCZ3301 0 μM** | 0.00 | 3.92% ± 1.68% | 6.71% ± 1.24% | 6.45% ± 1.08% | 32.42% ± 1.81% | 69.40% ± 2.46% | 74.76% ± 2.33% | 90.77% ± 1.40% |
| **DCZ3301 1 μM** | 5.01% ± 4.65% | 4.53% ± 1.98% | 7.05% ± 4.05% | 10.42% ± 0.68% | 18.57% ± 2.01% | 68.94% ± 2.36% | 74.93% ± 2.53% | 85.48% ± 3.42% |
| **DCZ3301 2 μM** | 7.80% ± 3.86% | 4.99% ± 2.18% | 7.30% ± 3.46% | 18.38% ± 3.77% | 16.66% ± 4.54% | 67.41% ± 2.83% | 75.48% ± 2.47% | 89.98% ± 2.66% |
| **DCZ3301 4 μM** | 12.39% ± 3.02% | 10.65% ± 0.74% | 21.79% ± 2.90% | 23.77% ± 3.70% | 45.90% ± 2.53% | 67.75% ± 3.12% | 75.62% ± 2.41% | 94.68% ± 2.06% |

**Supplementary Table 8**

Combination Index of Bortezomib Combination treatment with DCZ3301 in Bortezomib resistant cell line **RPMI-8226**.

|  | **Combination Index** |  |  |  |  |  |  |
| --- | --- | --- | --- | --- | --- | --- | --- |
|  | **Bortezomib 1 nM** | **Bortezomib 2 nM** | **Bortezomib 4 nM** | **Bortezomib 8 nM** | **Bortezomib 16 nM** | **Bortezomib 32 nM** | **Bortezomib 64 nM** |
| **DCZ3301 1 μM** | 1.82 | 1.44 | 1.66 | 1.82 | 0.77 | 1.23 | 1.53 |
| **DCZ3301 2 μM** | 1.56 | 2.07 | 1.62 | 2.31 | 0.87 | 1.25 | 1.54 |
| **DCZ3301 4 μM** | 1.22 | 1.38 | 1.82 | 1.12 | 0.98 | 1.33 | 1.39 |

**Supplementary Figure 1**


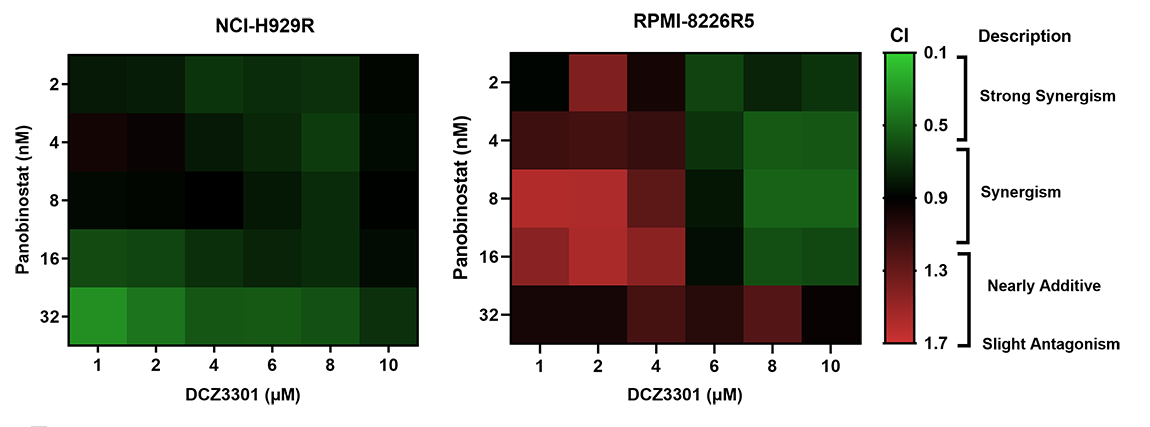


The heat map represents the CI value of DCZ3301 combined with panobinostat treatment in BTZ-resistant cell lines, NCI-H929R and RPMI-8226R5. CI values =1 always represents an additive effect, while CI values <1 and >1 indicate synergistic and antagonistic interactions, respectively. These results demonstrated that DCZ3301 has synergistic effect with panobinostat in BTZ-resistant MM cell lines.
